# Incidences of Herpes Zoster and Acne by Age in Japanese Patients With Atopic Dermatitis During Treatment With Baricitinib or Upadacitinib: A Single‐Centre Retrospective Study

**DOI:** 10.1111/exd.70100

**Published:** 2025-04-14

**Authors:** Ayu Watanabe, Masahiro Kamata, Yoshiki Okada, Yayoi Tomura, Yayoi Tada

**Affiliations:** ^1^ Department of Dermatology Teikyo University School of Medicine Tokyo Japan

**Keywords:** acne, atopic dermatitis, baricitinib, herpes zoster, JAK inhibitor, upadacitinib

**Abbreviations**
AD: atopic dermatitis
HZ: herpes zoster
PY: patient year(s)

1

Oral Janus kinase (JAK) inhibitors showed efficacy and effectiveness for moderate‐to‐severe atopic dermatitis (AD) in clinical trials and real‐world data [1, 2]. However, increased risks of herpes zoster (HZ) and acne are major concerns during treatment. Previous evidence revealed that elderly patients with rheumatoid arthritis were at a higher risk of developing HZ [3] and that younger AD patients were at a higher risk of developing acne [4], but their incidences by age in AD patients were unknown. We retrospectively investigated their incidences by age in Japanese AD patients.

All AD patients treated with baricitinib or upadacitinib in our department between January 2021 and May 2024 were included. The incidences of HZ and acne (per 100 patient years [PY]) were calculated. To ensure balanced representation across age groups, patients were categorised into age ranges: 12–14, 15–24, 25–34, 35–44 and 45 years and older. We counted acne as an adverse event when patients newly developed acne or when patients having acne at initiation of the drug showed exacerbation during the treatment with baricitinib or upadacitinib.

Data from 84 patients, of whom 42 were treated with baricitinib (female, 15; male, 27) and 42 with upadacitinib (10; 32), were analysed. The mean ages and standard deviations of patients treated with baricitinib or upadacitinib and of all patients were 33.8 ± 11.4, 28.9 ± 13.0 and 31.3 ± 12.4 years, respectively. Before receiving baricitinib, 9 patients were treated with dupilumab, 3 with cyclosporine and 1 with upadacitinib. Before receiving upadacitinib, 6 patients were treated with dupilumab and 13 with baricitinib. The total observation periods of patients treated with baricitinib and upadacitinib were 39.0 PY and 49.4 PY. With one exception, no patients had a history of HZ at the initiation of baricitinib or upadacitinib.

The incidences of HZ were 5.1, 8.1 and 6.8/100 PY in patients treated with baricitinib and upadacitinib, and total patients (Figure 1A). The odds ratio of upadacitinib against baricitinib was 2.1. HZ was not observed in patients aged 15–44 years receiving baricitinib or in those aged 12–24 years receiving upadacitinib. Patients over 45 years showed the highest incidences of HZ in each treatment group (29.1, 27.3 and 28.1/100 PY). The mean duration of baricitinib treatment at developing HZ was 4.5 ± 2.5 months, that of upadacitinib was 15.5 ± 11.4 and that of either drug was 11.8 ± 10.8. No evident tendency by age was observed. One in two patients who developed HZ during baricitinib treatment had received cyclosporine before initiating baricitinib, and the other had never received systemic therapy. Two in four patients who developed HZ during upadacitinib treatment had received dupilumab, and the others had received baricitinib before initiating upadacitinib.

**FIGURE 1:**
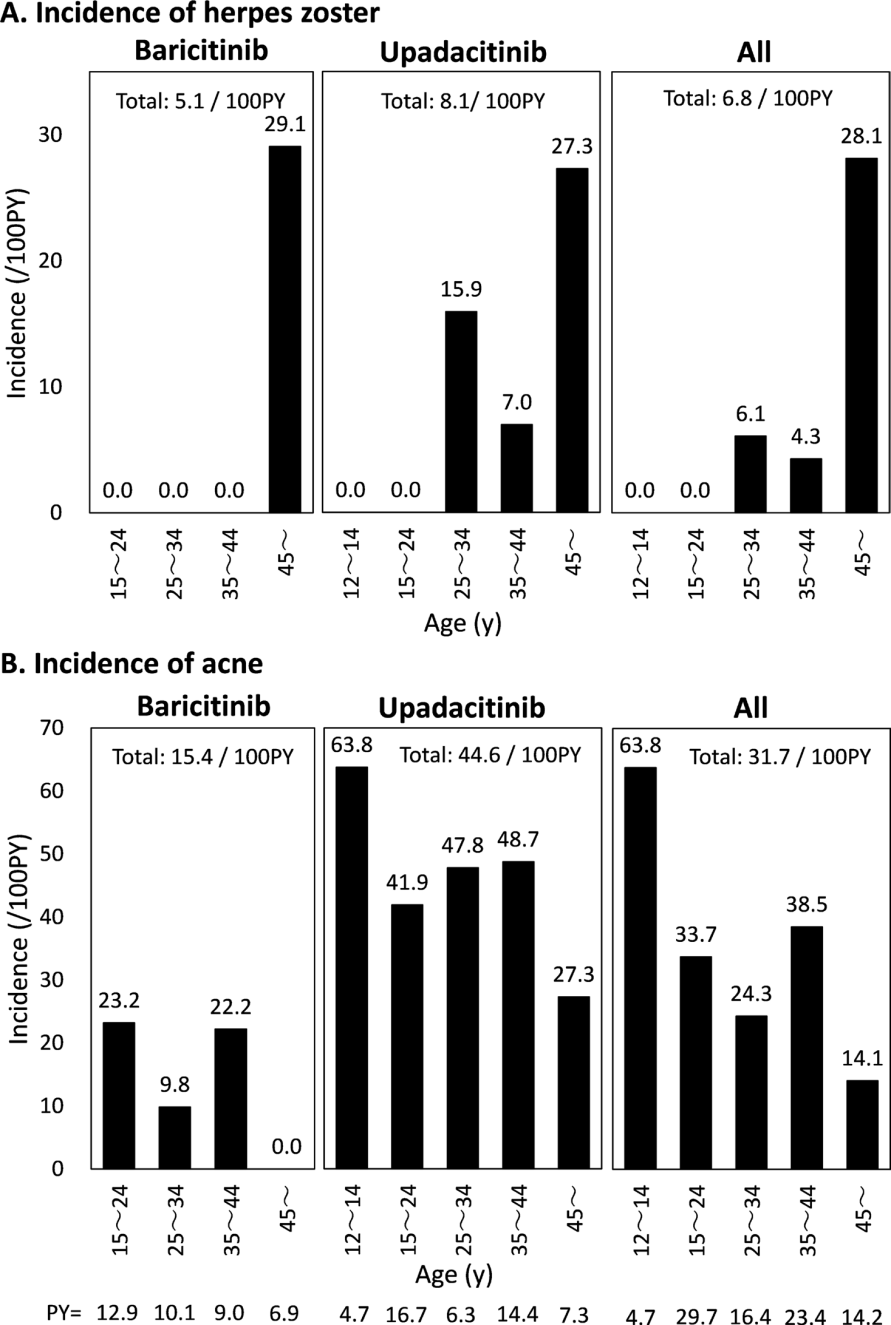
Incidences of herpes zoster (A) and acne (B) by age in patients with atopic dermatitis during treatment with baricitinib or upadacitinib. PY, patient years; y, year(s).

The incidences of acne were 15.4, 44.6 and 31.7/100 PY (Figure 1B). The odds ratio of upadacitinib against baricitinib was 4.59. Patients aged over 45 years showed the lowest incidences of acne in each group (0.0, 27.3 and 14.1/100 PY), whereas patients aged 12–14 years treated with upadacitinib showed the highest incidence of acne (63.8/100 PY). The mean duration of baricitinib treatment at developing acne was 5.9 ± 8.3 months, that of upadacitinib was 5.2 ± 5.5 and that of either drug was 5.4 ± 6.6. No evident tendency by age was observed. The severity of acne was mild or moderate in all the patients. Acne was successfully treated with topical agents in most patients.

The incidences of HZ and acne were higher in patients treated with upadacitinib than in those with baricitinib in our study, which is compatible with previously reported data [1]. Our data showed that HZ was not observed in patients aged 15–44 years receiving baricitinib or in those aged 12–24 years receiving upadacitinib, indicating that HZ is of least concern in AD patients with no history of HZ under 25 years of age and in those aged 25–44 years. Acne can be a concern in patients under 45 years of age, especially adolescents. The small number of analysed patients is one limitation of this study. Almost all the patients treated with baricitinib received 4 mg/day for most of the observation period. Most of the patients treated with upadacitinib received doses of 15 or 30 mg/day, according to their signs and symptoms. Therefore, incidences by dose could not be evaluated, which is another limitation. Data on vaccines for HZ are lacking, which is one of the limitations of this study.

Our study indicates the lowest concern being of HZ in AD patients with no history of HZ under 45 years for baricitinib and under 25 years for upadacitinib, which underscores the safety of JAK inhibitors in young patients. JAK inhibitors can be a good treatment option, especially for this population.

## Author Contributions

A.W., M.K., Y.O., Y. Tomura and Y. Tada performed the research. M.K. designed the research study. Y. Tada contributed essential reagents or tools. A.W. and M.K. analysed the data. A.W. and M.K. wrote the paper.

## Ethics Statement

This study was approved by the ethics committee of Teikyo University (22‐015) and was carried out under the principles of the Declaration of Helsinki.

## Consent

Informed consent was obtained in the form of opt‐out on the website. Patients who rejected it were excluded.

## Conflicts of Interest

M.K. received honoraria for lectures from AbbVie and Eli Lilly. Y.T. received grants for research unrelated to this study from AbbVie and Eli Lilly, and honoraria for lectures from AbbVie and Eli Lilly.

## Data Availability

Data are available on reasonable request, subject to privacy/ethical restrictions.
